# Numerical Simulation of 30% Efficient Lead-Free Perovskite CsSnGeI_3_-Based Solar Cells

**DOI:** 10.3390/ma15093229

**Published:** 2022-04-29

**Authors:** Hussein Sabbah

**Affiliations:** College of Engineering and Technology, American University of the Middle East, Kuwait; hussein.sabbah@aum.edu.kw

**Keywords:** solar cell, photovoltaics, thin films, SCAPS simulation, lead-free perovskite, power conversion efficiency, electron transport layer

## Abstract

A cesium tin–germanium triiodide (CsSnGeI_3_) perovskite-based solar cell (PSC) has been reported to achieve a high-power-conversion efficiency (*PCE* > 7%) and extreme air stability. A thorough understanding of the role of the interfaces in the perovskite solar cell, along with the optimization of different parameters, is still required for further improvement in *PCE*. In this study, lead-free CsSnGeI_3_ PSC has been quantitatively analyzed using a solar cell capacitance simulator (SCAPS–1D). Five electron transport layers (ETL) were comparatively studied, while keeping other layers fixed. The use of SnO_2_ as an ETL, which has the best band alignment with the perovskite layer, can increase the power conversion efficiency (*PCE*) of PSC by up to 30%. The defect density and thickness of the absorber layer has been thoroughly investigated. Results show that the device efficiency is highly governed by the defect density of the absorber layer. All the PSCs with a different ETL exhibit *PCE* exceeding 20% when the defect density of the absorber layer is in the range of 10^14^ cm^−3^–10^16^ cm^−3^, and degrade dramatically at higher values. With the optimized structure, the simulation found the highest *PCE* of CsSnGeI_3_-based PSCs to be 30.98%, with an open circuit voltage (*V_oc_*) of 1.22 V, short-circuit current density (*J_sc_*) of 28.18 mA·cm^−2^, and fill factor (*FF*) of 89.52%. Our unprecedented results clearly demonstrate that CsSnGeI_3_-based PSC is an excellent candidate to become the most efficient single-junction solar cell technology soon.

## 1. Introduction

Energy consumption is expected to increase, with the bulk of the demand coming from developing countries. Conventional fuel remains the greatest contributor to electricity generation worldwide. Producing energy from these resources takes a severe toll on our environment. Solar energy is one of the most abundant energy resources on Earth, as it is sustainable and inexhaustible. Solar cells are regarded as clean and sustainable sources of energy [1,2]. Coupled with an efficiency rating of greater than 15%, they are a great energy resource [3,4]. To date, conventional silicon (C–Si) solar cells enjoy a market share exceeding 90% of the overall photovoltaic market [5], due to silicon’s high stability and power conversion efficiency (PCE). Theoretically, the room-temperature power conversion efficiency of a single junction C–Si solar cell is limited to 32.33% [6]. Kaneka Corporation achieved a real-world solar cell with 26.7% conversion efficiency [7,8]. However, there are several drawbacks, namely high cost, difficult preparation conditions, serious environmental pollution [9], and the indirect nature of its electronic band gap, making it a relatively weak absorber of long wavelength sunlight [10].

A decade ago, lead halide Perovskite solar cells (PSCs) emerged as a breakthrough photovoltaic (PV) technology. These cells have acquired significant interest in research and industrial communities due to their high-power-conversion efficiency exceeding 25% [11,12,13], straightforward fabrication process, and relatively low cost [14,15]. The state-of-the-art certified PCE of PSC in single junction architectures exceeded 25% in less than 10 years [16,17], owing to its tunable direct bandgap, high absorption coefficient, low exciton binding energy, and high carrier mobility [18,19,20,21]. Although the perovskite community has put great efforts into addressing the peculiar physics and chemistry of these materials, and has achieved remarkable results, the toxicity of lead associated with the lifecycle of these PSCs is a major concern, hindering their commercialization and future large-scale applications. To conquer this issue, scientists have devoted huge efforts to substitute lead with non-toxic metals such as tin, bismuth, and germanium [22,23,24,25].

Tin-based halide perovskites, such as CsSnI_3_, CH_3_NH_3_SnI_3_ (MASnI_3_), and HC(NH_2_)_2_SnI_3_ (FASnI_3_), have been investigated. The latter two perovskites have intrinsically low stability [26,27]. The all-inorganic lead-free CsSnI_3_ perovskite could be a promising alternative to Pb-based light harvesting materials, due to its improved photoelectric characteristics such as a high absorption coefficient, small exciton binding energy, high carrier mobility, etc., and is currently the strongest candidate [28,29,30,31]. Recently, several studies showed that the substitution of the organic cation by cesium (Cs) in the perovskite structure could significantly enhance its thermal stability and outdoor/ambient device performance [32,33]. Among all lead-free all-inorganic PSCs, CsSnI_3_ recorded the highest PCE of 10.1% [34,35]. Min Chen et al. [36] proposed a perovskite absorber material CsSnGeI_3_ that shows superior performance and exceptional air stability over its pure counterparts CsSnI_3_ and CsGeI_3_. The CsSnGeI_3_-based device shows a PCE of 7%.

This study aims to suggest possible optimization routes for efficiency improvements of the CsSnGeI_3_ perovskite solar cell, by analyzing various device parameters using a solar cell capacitance simulator (SCAPS-1D) [37]. Different types of simulation software exist, to study the perovskite solar cell performance via computational investigation, such as SILVACO ATLAS, AMPS, COMSOL, and SCAPS [38,39]. Among them, SCAPS presents unique advantages, such as the ability to simulate up to seven different layers with non-routine measurements (C-V, C-f). It can calculate many parameters such as spectral response, energy bands, ac characteristics, *J−V* curve, and defect density, by just solving three basic semiconductor equations. It is user friendly and may be executed in both dark and light atmospheres [40,41,42]. Several studies confirm the good agreement of the simulation results with the experimental data, indicating the reliability of SCPAS software [43,44].

This work focuses on engineering the interface ETL/perovskite absorber layer. Optimizing the interfaces in the PCSs is essential for enhancing the photovoltaic performance. In this study, different materials were investigated as ETL, mainly TiO_2_ and SnO_2_. TiO_2_ is the most common ETL for the fabrication of PSC [41]. On the other hand, SnO_2_ has high electron mobility and keeps better band alignment with the CsSnGeI_3_ absorber layer, which is supportive for electron extraction [45,46]. Cuprous oxide Cu_2_O was used as the hole transport layer (HTL) due to its low cost, and it is widely used in solar energy materials, as a promising inorganic HTL for PSC applications [47]. The impact of the defect density and the thickness of the absorber layer on the overall performance of the proposed device was studied. For the first time, we show that an optimum CsSnGeI_3_ device can have a simulated power conversion efficiency PCE of 30%.

## 2. Materials and Methods

In this numerical modeling, a comprehensive study has been performed on CsSnGeI_3_, as the light harvesting material using SCAPS, which is 1D solar cell simulation software [47]. The software is specially designed to simulate multilayer solar cells, up to seven layers. SCAPS permits the calculation and observation of many electrical characteristics and parameters including current-density (J−V) curve, hetero-junction energy band structure, quantum efficiency (QE), open circuit voltage $V_{oc}$, short circuit $J_{sc}$, current density, power conversion efficiency (PCE), fill factor (FF), etc. The algorithm of SCPAS software is developed to solve the Poisson equation (Equation (1)) and the continuity equation of both charge carriers, electron (Equation (2)), and hole (Equation (3)).

(1) $$\frac{d}{dx}\left(-\varepsilon \left(x\right)\frac{d\psi }{dx}\right)=q\left[p\left(x\right)-n\left(x\right)+N_{D}^{+}\left(x\right)-N_{A}^{-}\left(x\right)+p_{t}\left(x\right)-n_{t}\left(x\right)\right]$$

(2) $$\frac{dp_{n}}{dt}=G_{p}-\frac{p_{n}-p_{n0}}{\tau_{p}}+p_{n}\mu_{p}\frac{d\xi }{dx}+\mu_{p}\xi \frac{dp_{n}}{dx}+D_{p}\frac{d^{2}p_{n}}{dx^{2}}$$

(3) $$\frac{dn_{p}}{dt}=G_{n}-\frac{n_{p}-n_{p0}}{\tau_{n}}+n_{p}\mu_{n}\frac{d\xi }{dx}+\mu_{n}\xi \frac{dn_{p}}{dx}+D_{n}\frac{d^{2}n_{p}}{dx^{2}}$$

**Figure 1:**
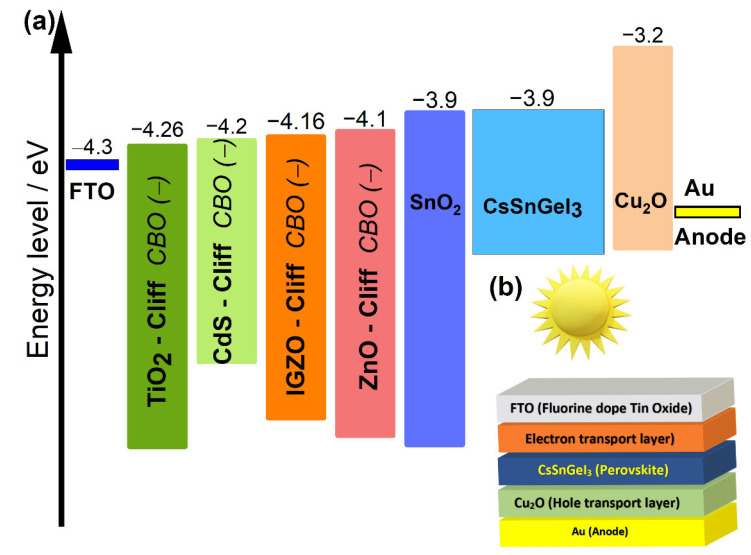
(**a**) Band alignment between ETL materials and CsSnGeI_3_ perovskite. (**b**) Schematic diagram of lead-free CsSnGeI_3_-based PSC structure.

The device simulation is carried out in the n−i−p configuration of FTO/ETL/CsSnGeI_3_/Cu_2_O/Au, which is represented in Figure 1b. It was performed at 300 K under a standard illumination of $1000\;W/m^{2}$ and an air mass of AM 1.5G. The absorber layer is sandwiched between the HTL and ETL layers. Fluorine-doped tin oxide (FTO) is used as a front contact and gold (Au) as back metal contact. HTM Cu_2_O is used for every structure, while five different ETLs are alternately used, and their performances are compared. The ETL includes: SnO_2_, ZnO, IGZO, CdS, and TiO_2_. Figure 1a shows the energy level diagram of the corresponding materials utilized in the device structure.

Table 1, Table 2 and Table 3 show the list of values extracted from theories, experiments, and the literature [48,49,50,51,52,53,54,55,56,57]. The parameters have been considered, while forming a base configuration to start with the simulation process. Different properties, such as the thickness and defect density of the absorber layer, have been varied to obtain an optimized result and to study their impacts on the device performance. Initially, the thickness of FTO $\left(400\;\mathrm{nm}\right)$, ETL $\left(50\;\mathrm{nm}\right),$ and Cu_2_O $\left(350\;\mathrm{nm}\right)$ were optimized for high PCE, as mentioned in Table 1.

## 3. Results and Discussions

### 3.1. Effect of ETLs on Solar Cell Performance

**Figure 2:**
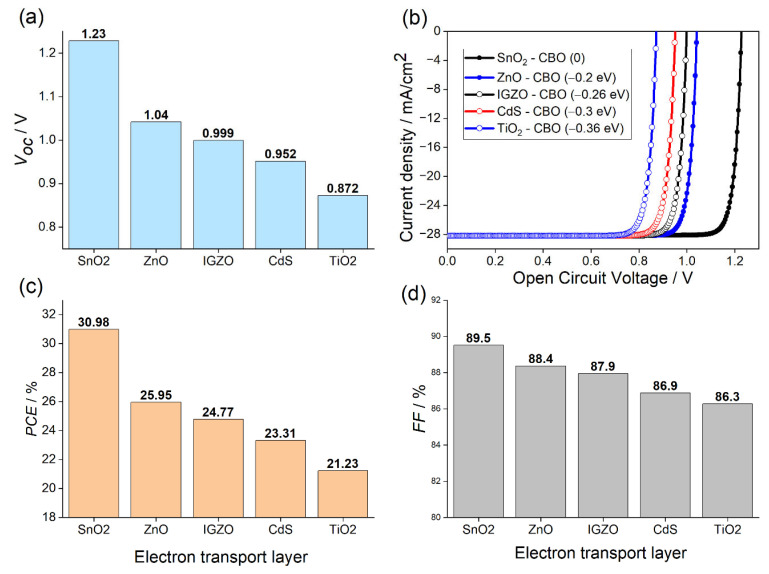
Variation in (**a**) $V_{oc}$, (**b**) J−V characteristics, (**c**) PCE, and (**d**) FF for different ETL materials simulated in PSC.

The n−i−p PSC structures for three different ETL are analyzed for the same absorber (CsSnGeI_3_) layer thickness, 1.5 μm, and the same absorber bulk defect densities, $1\times 10^{15}\;{\mathrm{cm}}^{-3}$. It should be mentioned that these values are selected and fixed after optimizing the device performance. The thickness and the defect concentration of the perovskite layer were investigated and varied, until the optimal performance of the solar cell is found. The J−V characteristics for the five structures are shown in Figure 2a, while the open circuit voltage ($V_{oc}$), performance cell efficiency (PCE), and fill factor (FF) are plotted, respectively, in Figure 2b–d. Figure 2b reveals that the five simulated cells exhibit the same current level of $28\;\mathrm{mA}\cdot {\mathrm{cm}}^{-2}$.

Surprisingly, all ETLs had superior performance over TiO_2_, the most commonly used ETL in perovskite studies due to its mesoscopic structure. It is observed that the PSC with SnO_2_ acquired the highest PCE  of 30.9% and $V_{oc}$ of 1.22 V. The most credible reason of this behavior is the excellent band alignment with the CsSnGeI_3_ layer shown in Figure 1a, where there is no conduction band offset (CBO) ($\Delta E_{c}=0$) at the SnO_2_/CsSnGeI_3_ interface. In fact, CBO is the energy difference $\Delta E_{c}$ between the CB level of ETL and that of the perovskite. When the CB level of ETL is lower than that of the perovskite, $\Delta E_{c}$ will be negative and an energy cliff will be formed at the ETL/perovskite interface. An energy cliff structure does not hinder the photo-generated electron flow, but it may cause the interfacial recombination to occupy the main position of recombination of the PSC device [58,59]. When the energy cliff is formed, there is no potential barrier for electrons at the ETL/perovskite interface. The energy barrier that the electron transport must overcome from the conduction band of the ETL to the interface ETL/perovskite is $\Delta E_{c}+qV_{D}$, here q is the charge of the carrier and $V_{D}$ is the built-in potential. Since $\Delta E_{c}$ is negative for all devices, except the one with SnO_2_ ETL, the electrons in the ETL layer can easily cross the barrier to attain the interface. The cliff formation can improve the accumulation near to ETL/perovskite, which in turn enhances the recombination in this area by back-transfer to the interface of heterojunction.

Under open-circuit conditions, the interfacial recombination mainly occurs through the pathways of back-transfer recombination, where the electrons that have been injected into the FTO electrode, and the free holes in the absorber recombine through the deep level defect at the ETL/absorber interface, which then result in a serious $V_{oc}$ deficit. Then, the PCE drops, which has been confirmed by our results plotted in Figure 2a,c. They exhibit that the $V_{oc}$ as well as the PCE dramatically decrease with the increase in the absolute value of $\Delta E_{c}$. It was observed in Figure 2d that FF is improved with devices having a larger $V_{oc}$. The solar cell with the benchmark material TiO_2_ ETL yields the lowest PCE of 21.23%, *V_oc_* of 0.872 V, and *FF* of 86.3%, owing this to the largest CBO among all the tested materials ($\Delta E_{c}=-0.36\;\mathrm{eV}$), comparable to the best performance obtained in recent studies on similar perovskite [60].

Finally, it can be concluded that the band alignment plays one of the key roles for having higher PCE, $V_{oc}$*,* and FF. Thus, SnO_2_ is finally selected as the optimal ETL material for the proposed lead-free PSC.

### 3.2. Effect of Absorber Layer Defects Density $N_{t}$

**Figure 3:**
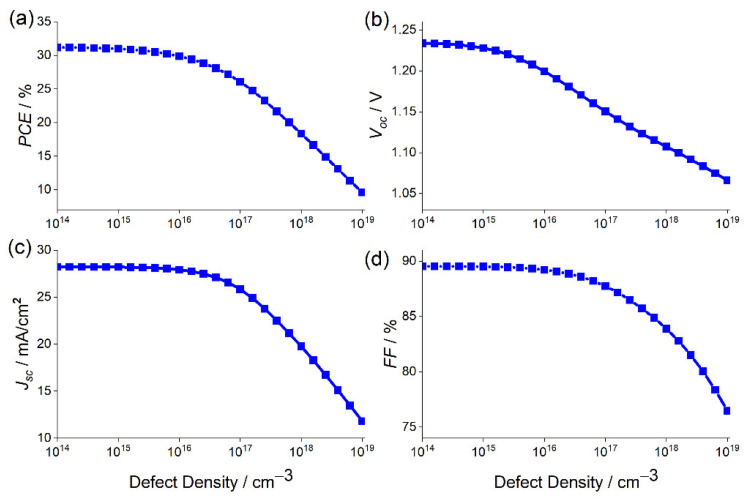
Photovoltaic characteristics of (**a**) PCE, (**b**) $V_{oc}$, (**c**) $J_{sc}$ and (**d**) FF as a function of the defect density of the absorber layer.

Although the interface engineering and the control at the ETL/perovskite interface is crucial for achieving a high-efficiency solar cell device, the morphology and quality of the light absorber film have been recently recognized as a major factor for determining PSC performance [61], given that the photoelectrons are mainly generated in this layer. Thus, to further enhance the PSC performance with SnO_2_ ETL and to determine the influence of absorber defect density on cell performance, the defect density of the perovskite layer has been considered. The defect density values simulated in this study range from $10^{14}{\mathrm{cm}}^{-3}$ to $10^{19}\;{\mathrm{cm}}^{-3}$, based on values from previous studies on lead [62,63]. As shown in Figure 3a–d, when the defect density is below $N_{t}=10^{16}\;{\mathrm{cm}}^{-3},$ the photovoltaic response of the cell remains almost unchanged, except $V_{oc},$ which starts to decrease. The PSC performance properties start to deteriorate when the defect density exceeds $10^{16}{\;\mathrm{cm}}^{-3},$ until reaching their lowest performance values of $V_{oc}=1.06\;V$, $J_{sc}=11.79\;\mathrm{mA}\cdot {\mathrm{cm}}^{-2}$, PCE=9.61%, and FF=76.46%.

**Figure 4:**
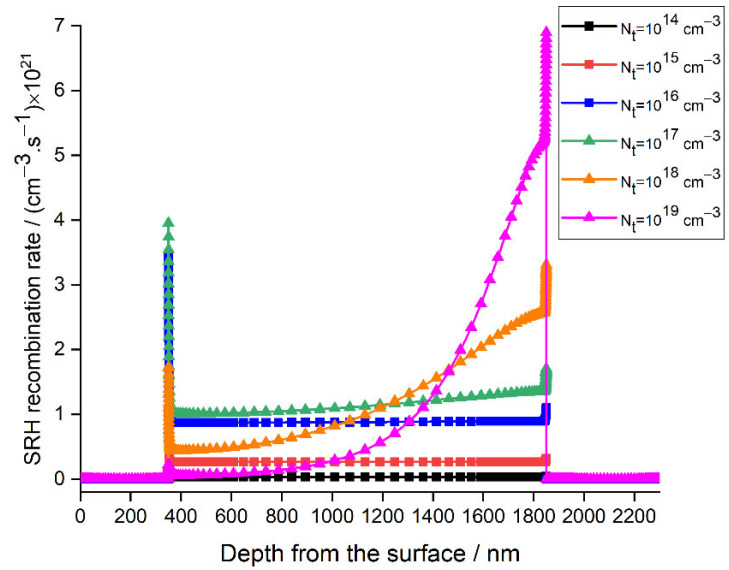
Effect of the absorber layer defect density on the recombination rate with depth from the surface.

The perovskite defect density effect can be explained by the Shockley–Read–Hall (SRH) recombination model [64,65]. In this work, to clearly understand the defect density effect of the absorber on the performance of the device, the variation of the SRH recombination rate with depth from the surface for different values of defect density was studied and depicted in Figure 4. It is detected that the recombination rate increases with the increase in the defect density. Consequently, $V_{oc}$ and PCE decrease, as shown in Figure 3a,b. It can be observed that the recombination rate not only increases in the absorber layer between 350 nm and 1850 nm but also at the perovskite/ETL interface, reaching a maximum of $7\times 10^{21}{\;\mathrm{cm}}^{-3}\cdot s^{-1}$.

According to the SRH model, the recombination rate can be expressed by:

(4) $$R^{SRH}=\frac{\tau_{n,p}^{-1}\left(n\cdot p-n_{i}^{2}\right)}{n+p+2n_{i}coh\left(\frac{E_{t}-E_{i}}{KT}\right)}$$

where, $\tau_{n,p},n,p,n_{i},E_{i,}$ and $E_{t}$ represent the lifetime of charge carriers, electron density, hole density, intrinsic density, intrinsic energy level, and energy level of the trap defects, respectively.

The lifetime of charge carriers $(\tau_{n,p)}$, the diffusion coefficient $\left(D\right)$, and the diffusion length of charge carrier $\left(L_{D}\right)$ are represented in the following equations:

(5) $$\tau_{n,p}=\frac{1}{\sigma_{n,p}v_{th}N_{t}}$$

(6) $$L_{D}=\sqrt{D\tau }$$

(7) $$D=\frac{\mu \cdot K_{B}T}{q}$$

where μ is the mobility of charge carriers, T is the temperature in Kelvin, $K_{B}$ is the Boltzmann constant, and q the magnitude of the charge.

It can be deduced from Equations (4)–(7) that as the defect density in the perovskite layer increases, the carrier lifetime decreases, while the charge recombination will increase, as confirmed in Figure 4, leading to a drop in $V_{oc}$ and $J_{sc}$. Moreover, when the doping density is comparable or lower than that of the defect density in the absorber, the device becomes semi-insulating and the desired p-n junction will not be formed [66], resulting in inferior cell performance. This scenario was confirmed by the current study, wherein the doping density for CsSnGeI_3_ is $10^{17}\;{\mathrm{cm}}^{-3},$ and the performance of the device starts to worsen when the defect density exceeds $10^{16}{\;\mathrm{cm}}^{-3}$.

It can be observed from Equation (7) that the diffusion coefficient is proportional to the mobility of charge carrier, the electron mobility ($974{\;\mathrm{cm}}^{2}/V\cdot s$), and hole mobility ($213{\;\mathrm{cm}}^{2}/V\cdot s$), being larger for CsSnGeI_3_. Due to the large value of mobility, the diffusion length is high, and that is why our simulated PSC shows a high efficiency of 30.98% with $N_{t}=10^{15}{\;\mathrm{cm}}^{-3}$. Thus, to achieve optimum PSC performance, the defect density in the absorber layer has to be significantly reduced. Experimental studies have proven that the fabrication of the tin-based perovskite device, with defect density as low as $N_{t}=10^{15}{\;\mathrm{cm}}^{-3},$ is completely feasible [67], so we set this defect density as an optimized value. By fixing $N_{t}=10^{15}{\mathrm{cm}}^{-3}$, the cell performance reaches its maximum performance, attaining a $J_{sc}$ of $28.19\;\mathrm{mA}\cdot {\mathrm{cm}}^{-2}$, PCE of 30.98%, FF of 89.52%, and $V_{oc}$ of 1.22 V. Further decreasing the defect density from $N_{t}=10^{15}\;{\mathrm{cm}}^{-3}$ to $N_{t}=10^{14}\;{\mathrm{cm}}^{-3}$ has a negligible effect. The obtained performance is found to be largely better than the previously published results for lead-free perovskite solar cells [60].

### 3.3. Influence of the Absorber Layer Thickness

**Figure 5:**
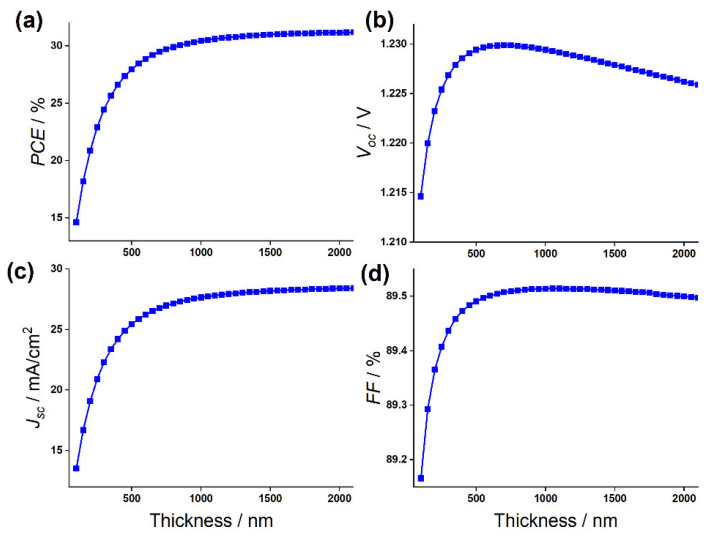
Change in (**a**) PCE, (**b**)$\;V_{oc}$, (**c**) $J_{sc},$ and (**d**) FF against the CsSnGeI_3_ absorber layer thickness variation.

The thickness of the light-absorbing layer plays a crucial role in optimizing the solar cell performance. It should be chosen as high to maximize the current density, but not too much, as it minimizes the reverse saturation current. The influence of the absorber layer thickness on the device photovoltaic outputs has been studied, by varying it from 0.1 μm to 2 μm while maintaining all the other parameters as given in Table 1. Figure 5a–d depicts the PSC performance statistics, including $V_{oc}$, $J_{sc}$, PCE, and FF. It can be observed that the effect of absorber layer thickness is minimal. A FF of 89.16% and 89.50% are obtained at an absorber layer thickness of 0.1 μm and 0.6 μm, respectively, then it saturates. The value of $V_{oc}$ is obtained to be 1.214 V at 0.1 μm and increases up to a maximum of 1.230 V at 0.6 μm, then it decreases to 1.225 V at 0.6 μm. The decreases in $V_{oc}$ with thickness is attributed to the increase in the dark saturation current, which increases the recombination of the charge carrier. This dependency of the dark saturation current $J_{0},V_{oc},$ and $J_{sc}$, is given by [68]:

(8) $$V_{oc}=\frac{K_{B}T}{q}Ln\left[\frac{J_{sc}}{J_{0}}+1\right]$$

It is seen in Figure 5c that $J_{sc}$ increases linearly, up to an absorber thickness of 1 μm, then it almost saturates with an increasing absorber thickness. The $J_{sc}$ of $13.51\;\mathrm{mA}\cdot {\mathrm{cm}}^{-2}$ and $28.45\;\mathrm{mA}\cdot {\mathrm{cm}}^{-2}$ are estimated for a thickness of 0.1 and 2 μm, respectively. The great enhancement of $J_{sc},$ with an increase in the absorber thickness, is attributed to the generation of more electron-hole pairs in the perovskite, leading to an efficiency enhancement. The trend of PCE as a function of CsSnGeI_3_ was strongly correlated with the trend of $J_{sc}$, and PCEs of 14.63% and 31% are estimated for a thickness of 0.1 and 2 μm, respectively.

**Figure 6:**
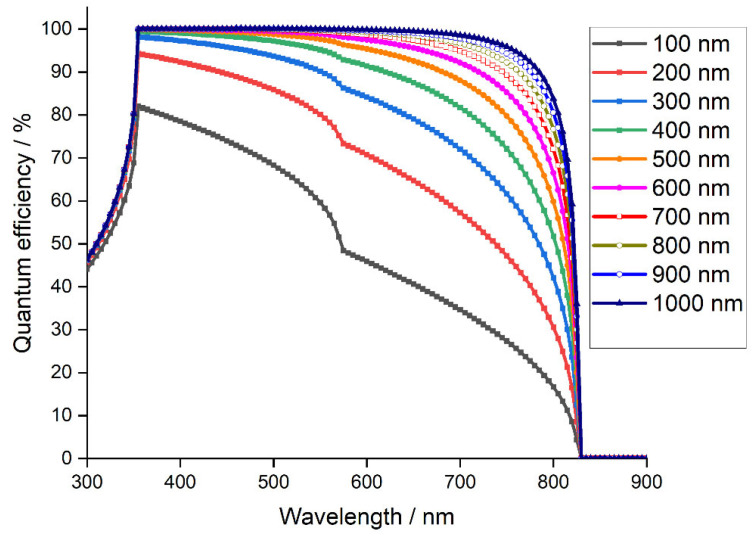
Quantum efficiency of the simulated device with absorber layer thickness.

Figure 6 depicts quantum efficiency vs. wavelength for various absorber thicknesses ranging from 0.1 μm. to 1 μm. Quantum efficiency indicates to what extent a solar cell can collect carriers from incident photons of a given energy. It can be observed that the decrease in absorber thickness leads to reduced photon absorption at a longer wavelength. This is due to fewer photogenerated electron-hole pairs inside the absorber layer. In addition, at wavelengths larger than 830 nm, quantum efficiency falls to zero as light is not absorbed below the bandgaps, at longer, low energy wavelengths. A loss of quantum efficiency can be observed for wavelengths below 350 nm for all simulated solar cells. This is because the light is mainly absorbed by the FTO substrate and SnO_2_ layer.

The larger *QE* is reached with an absorber thickness of 1 μm. Ultimately, the best performing device has a perovskite layer 1.5 μm thick with $J_{sc}$ of $28.19\;\mathrm{mA}\cdot {\mathrm{cm}}^{-2}$, PCE of 30.98%, FF of 89.52%, and $V_{oc}$ of 1.22 V.

## 4. Conclusions

In this work, a novel lead-free CsSnGeI_3_-based perovskite solar cell has been thoroughly investigated. A normal n-i-p planar structure of FTO/ETL/CsSnGeI3/Cu2O/Au is numerically simulated and analyzed using SCAPS-1D simulation software. The impact of various ETL on the device performance was studied to get the highest possible efficiency. It has been demonstrated that a PSC with SnO_2_ ETL has, by far, the best performance, owing to its excellent band alignment with the perovskite layer. Furthermore, the photovoltaic performance of the cell has been optimized by tuning two major factors: CsSnGeI_3_ defect density and absorber layer thickness. Our study explained the significant effect of these two factors on the electrical parameters of the PSC. The results revealed that the optimal light absorber thickness and the optimal absorber defect density were 1.5 μm and $10^{15}\;{\mathrm{cm}}^{-3}$, respectively. Reducing the absorber defect density significantly enhances the PCE to reach an unprecedented result of almost 31%. Thus, future research should focus on refining the device fabrication techniques. Our novel results could provide a viable path to fabricate cost-effective, highly efficient, and stable CsSnGeI_3_-based perovskite solar cells.

## Figures and Tables

**Table 1:** Electrical and optical properties used in simulation of CsSnGeI_3_-based perovskite solar cell.

Parameters	FTO [56]	SnO_2_ (ETL) [54,55]	CsSnGeI_3_ (Absorber) [36]	Cu_2_O [48,50]
Thickness (µm)	0.4	0.05	1.5	0.350
Bandgap $E_{g}$(eV)	3.5	3.6	1.5	2.170
Electron Affinity χ(eV)	4.3	3.90	3.90	3.2
Dielectric permittivity	9	8	28	7.11
CB effective density of states $\left({\mathrm{cm}}^{-3}\right)$	$2.2\times 10^{18}$	$3.16\times 10^{18}$	$3.1\times 10^{18}$	$2\times 10^{17}$
VB effective density of states $\left({\mathrm{cm}}^{-3}\right)$	$1.8\times 10^{19}$	$2.5\times 10^{19}$	$3.1\times 10^{18}$	$1.1\times 10^{19}$
Electron mobility $\left({\mathrm{cm}}^{2}/V\cdot s\right)$	20	15	974	20
Hole mobility $\left({\mathrm{cm}}^{2}/V\cdot s\right)$	10	0.1	213	80
Donor Concentration $N_{D}\left({\mathrm{cm}}^{-3}\right)$	$1\times 10^{18}$	$1\times 10^{17}$	0	$1\times 10^{7}$
Acceptor concentration $N_{A}\left({\mathrm{cm}}^{-3}\right)$	0	0	$1\times 10^{19}$	$1\times 10^{18}$

**Table 2:** Electrical and optical properties of different ETL materials.

Parameters	ZnO [51,52]	IGZO [51,52]	CdS [53,57]	TiO_2_ [49]
Thickness (µm)	0.05	0.05	0.05	0.05
Bandgap $E_{g}$(eV)	3.3	3.05	2.4	3.260
Electron Affinity χ(eV)	4.1	4.160	4.2	4.26
Dielectric permittivity	9	10	10	32
CB effective density of states $\left({\mathrm{cm}}^{-3}\right)$	$1\times 10^{19}$	$5\times 10^{18}$	$2.2\times 10^{18}$	$1\times 10^{19}$
VB effective density of states $\left({\mathrm{cm}}^{-3}\right)$	$1\times 10^{19}$	$5\times 10^{18}$	$1\times 10^{19}$	$1\times 10^{19}$
Electron mobility $\left({\mathrm{cm}}^{2}/V\cdot s\right)$	50	15	100	20
Hole mobility $\left({\mathrm{cm}}^{2}/V\cdot s\right)$	5	0.1	25	10
Donor Concentration $N_{D}\left({\mathrm{cm}}^{-3}\right)$	$1\times 10^{17}$	$1\times 10^{17}$	$2\times 10^{18}$	$1\times 10^{17}$
Acceptor concentration $N_{A}\left({\mathrm{cm}}^{-3}\right)$	0	0	0	0

**Table 3:** Defect density values inside the layers and at interface of the device.

Parameters	ETL	HTL	CsSnGeI_3_	HTL/CsSnGeI_3_	CsSnGeI3/ETL
Defect Type	Neutral	Neutral	Neutral	Neutral	Neutral
Capture cross section for electrons $\sigma_{n}\left({\mathrm{cm}}^{-2}\right)$	$1\times 10^{-15}$	$1\times 10^{-15}$	$1\times 10^{-15}$	$1\times 10^{-18}$	$1\times 10^{-15}$
Capture cross section for hole $\sigma_{p}\left({\mathrm{cm}}^{-2}\right)$	$1\times 10^{-15}$	$1\times 10^{-15}$	$1\times 10^{-15}$	$1\times 10^{-16}$	$1\times 10^{-15}$
Energetic distribution	Single	Single	Gaussian	Single	Single
Energy level with respect to $E_{v}$$(\mathrm{above}\;E_{v}$) (eV)	0.6	0.650	0.6	0.6	0.6
Characteristic energy (eV)	0.1	0.1	0.1	0.1	0.1
Total density $N_{t}\left({\mathrm{cm}}^{-3}\right)$	$1\times 10^{15}$	$1\times 10^{14}$	$1\times 10^{15}$	$1\times 10^{12}$	$1\times 10^{11}$

## Data Availability

Not applicable.
